# A pilot study of an in-vitro bovine trachea model of the effect of continuous positive airway pressure breathing on airway surface liquid

**DOI:** 10.1186/1475-925X-13-12

**Published:** 2014-02-06

**Authors:** David E White, Roy J Nates, Jim Bartley

**Affiliations:** 1 School of Engineering, Auckland University of Technology, Auckland, New Zealand; 2 Department of Surgery, University of Auckland, Auckland, New Zealand

**Keywords:** Continuous positive air-pressure, Heated humidification, Airway surface liquid, Mucosal water flux, Nasopharyngeal side-effects, Upper airway

## Abstract

**Background:**

Continuous positive air pressure (CPAP) users frequently report troublesome symptoms of airway dryness and nasal congestion. Clinical investigations have demonstrated that supplementary humidification reduces these symptoms but the reason for their occurrence remains unexplained. Investigations using human computational air-conditioning models are unable to reproduce or quantify the apparent airway drying experienced during CPAP therapy. The purpose of this study was to determine whether augmented air pressures change overall mucosal airway surface liquid (ASL) water supply and, if so, the extent of this effect.

**Method:**

In an original in vitro experimental set up, maximal ASL supply was determined in whole bovine trachea when exposed to simulated tidal breathing stresses over a range of air pressures.

**Results:**

At ambient pressure, the maximal supply of ASL was found to compare well to previously published data (31.2 μl/cm^2^.hr). CPAP pressures from 5 cm H_2_O above ambient were found to reduce ASL supply by 22%. Statistical analysis (n = 8) showed a significant difference existed between the ambient and CPAP results (p < 0.0001), and that there was no significant variation between all pressurized results (p = 0.716).

**Conclusions:**

These findings provide preliminary data that ASL supply is reduced by CPAP therapy which may explain the airway drying symptoms associated with this therapy.

## Background

Many obstructive sleep apnea (OSA) patients receiving continuous positive airway pressure (CPAP) therapy report troublesome symptoms associated with airway drying. These are most commonly experienced in the nose and range from dryness, crusting, and congestion to sneezing, rhinorrhea and itching [1-5]. Heated humidification is used to manage these symptoms and improve patient comfort [6], however treatment compliance is not necessarily improved [7]. Despite the common use of supplementary humidification, the reason(s) why breathing ambient air at slightly elevated pressures might cause nasal drying are currently inconclusive.

Previously, nasal symptoms when using a CPAP nasal mask have been attributed to unidirectional airflow created by mouth leaks [8,9]. This situation potentially creates high rates of nasal air inflow, which are sustained throughout the breath cycle. Nasal mucosal rewetting through condensation during exhalation is reduced. Although mouth leaks could potentially dry the airway mucosa, symptoms of airway drying still occur when leaks are absent [10]. Nasal mucosal drying may also cause nasal congestion through the release of vasoactive amines and leukotrienes. These inflammatory mediators increase superficial mucosal blood flow, which leads to engorgement of deeper capacitance vessels that cause increased nasal resistance to airflow [11]. With nasal congestion being frequently cited as the main cause of poor adherence to CPAP therapy [6], mucosal drying is commonly relieved through the use of supplementary humidification in an attempt to improve treatment compliance.

The airway surface liquid (ASL) lining the upper respiratory tract has an essential role in heat and moisture exchange, as well as having an important role in airway defense. The ASL lining the respiratory tract comprises two distinct layers [12]. The airway lumen is lined with a mucus layer containing mucins secreted from goblet cells and glands. This layer is designed to trap and clear inhaled foreign material. Both mucin composition and “hydration” state influence the viscoelastic properties of this layer, which consists largely of water (97%). Behind the mucus is the pericilial layer (PCL) in which the cilia beat, causing the mucus layer to be transported for clearance. The PCL was considered a thin, low-viscosity, aqueous layer, however recently this layer has been shown to have a macromolecular glycoconjugate structure with a higher density than the mobile gel layer above [13]. This dense network is attached to the epithelial surface.

It has previously been suggested that symptoms of airway drying during spontaneous CPAP breathing may be caused by a reduction in the overall water movement from the mucosa to the ASL [14]. This could occur as a result of pressure suppression of cellular purinoceptor activation. Normally the NaCl concentration within the ASL is isotonic with plasma. Changes in Na^+^ or Cl^-^ transport are important in the regulation of the ASL hydration state [15]. These changes are achieved through ionic fluid channel activity, controlled via the activation of P1 and P2 purinoceptors. These purinoceptors, located in the apical membrane of airway epithelial cells, also influence ATP concentrations within the ASL [16]. Cyclic compressive and shear stresses elicited during normal tidal breathing increase the rate of ATP release [17]. This inhibits Na^+^ absorption and stimulates Cl^*-*^ and K^+^ secretion. Significantly, static, non-oscillatory stress does not stimulate ATP release or ion transport [18]. The constant low-level positive airway pressures used in CPAP therapy could potentially reduce ATP release to basal levels, thus reducing Cl^-^ and K^+^ fluid transport during a period of potentially high ASL water demand. This may occur as a consequence of the mucosa experiencing a substantial increase in stress elicited by CPAP pressure forces. Reduction in ASL hydration through suppressed mucosal ASL flux could explain nasal symptoms associated with airway drying during CPAP breathing.

A recent computational model of the human airway has predicted a cyclic change in ASL height during tidal breathing ambient air [19]. This study relied on maximal cellular water flux levels determined at ambient pressure [20,21] and did not predict the occurrence of airway drying.

This investigation is an aspect of a larger program aimed at understanding and modeling the nasal breathing cycle with respect to CPAP conditions. It tests the hypothesis that the low-level positive airway pressures used in CPAP therapy could reduce the ability of respiratory mucosa to humidify inhaled air as a result of reduced ASL supply from the airway mucosa. This pilot study demonstrates the effect CPAP breathing has on tracheal mucosal water flux, given that a reduction in mucosal ASL supply could lead to mucosal drying.

## Methods

### Dissection and mounting

**Figure 1:**
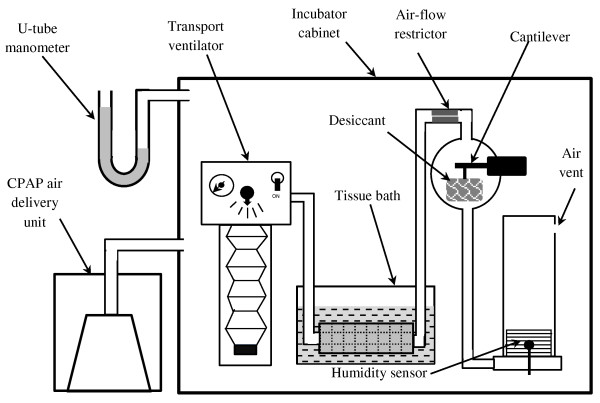
**Schematic representation of the apparatus set up within the incubator.** Bellows of transport ventilator shown in extended position and slave bellows in compressed state. Exhalation phase occurs when transport ventilator bellows compress. Bovine trachea immersed in physiological salt solution within tissue bath. Pressure augmentation supplied by CPAP air delivery unit is connected to sealed incubator cabinet.

An intact length of tracheal tissue, ranging in length from 220 to 300 mm and diameter from 32 to 38 mm, was cut and used in each experiment. A simple visual inspection of the wetted internal surface for each specimen confirmed the presence of an intact ASL layer. One tracheal end was attached to a suitable diameter glass tube by PVC plastic cable ties to ensure a secure gas-tight seal. The tracheal sample was then passed through a stainless steel spring (medical grade 6061); this provided an external distributed mass that resisted buoyancy forces and ensured the trachea remained immersed in the PSS contained within an open glass tissue bath. A second glass tube, selected again to be a tight fit, was inserted into the other tracheal end where it was also attached securely. The inside of the tissue was then rinsed with 100 ml of PSS. Any surplus PSS was allowed to drain from the tracheal sample and glass tubing for 3 minutes before the whole trachea and glass tube assembly was immersed in PSS contained within the glass tissue bath. Figure 1 shows a diagrammatic representation of the system which contained a fixed volume of air that was moved between two elastomeric bellows assemblies. The master driving bellows, actuated by a modified transport ventilator, was inverted and weighted at the free end to ensure that it returned to the extended position when not being driven. The slave bellows assembly, in opposition, were mounted in the upright position so that it returned by gravitational forces to the compressed position. Both bellows alternatively expanded and contracted as the trapped air volume was shuttled backwards and forwards during actuation by the transport ventilator, simulating tidal airflow. When assembled, 2 liters of ambient air was trapped within the closed system.

An orifice, located near the slave bellows assembly, was designed to provide flow resistance similar to the human nose [22]. During the inhalation phase of the simulated breath cycle, tracheal epithelial cells were exposed to a sub ambient static air pressure distribution and unidirectional shear stress. Conversely, during the exhalation phase, trachea epithelial cells experienced an above ambient pressure distribution with shear stress in an opposite direction to that occurring during inhalation.

The tissue mounting system containing PSS was placed in a water tank pre-heated by an immersion heater located within an incubator. The test apparatus was then assembled within the incubator space which provided the ambient air conditions for the system. The transport ventilator was then configured to deliver a tidal volume of 1 liter at a frequency of 6 breaths per minute, before the incubator door was closed. The temperature within the incubator space was then gradually raised over a 25 minute period from ambient to 37°C.

### Pressure augmentation

When required, pressure augmentation within the incubator space was provided by a CPAP air delivery unit (Fisher & Paykel Healthcare HC 234) connected to an air entry point within the incubator cabinet. Incubator pressure varied in increments of 5 cm H_2_O from ambient pressure up to 15 cm H_2_O. This pressure was transmitted to both sides of the trachea through the open tissue bath and vented slave bellows assembly.

The static air pressure augmentation experienced by the tissue and the complete test apparatus was measured using a manometer. Data acquisition was commenced after the complete system had stabilized at a temperature of 37°C for a 10 minute period.

### Tissue viability

To confirm pre-test trachea tissue viability, an unused portion of the trachea tissue was mounted in a silicon filled petri dish, before being bathed in PSS. A small drop of Indian ink was then released from a 1 ml Pasteur pipette onto the tracheal tissue surface and observed under a microscope at 100× magnification. Small iron particles present within the Indian ink were seen transported by coordinated motile cilia beating. The presence of a coherent mucociliary transport velocity (MTV) occurring across the mucosal surface was used to confirm tissue viability. Both still and moving images of the iron particle transport were recorded. Post experimentation testing of tissue viability was undertaken on tissue used in the experiment, approximately 7 hours after the experiment commenced. In all cases, this confirmed the tissue had remained viable throughout the duration of the experiment.

### Water demand and measurement

A glass sphere containing an open weave fabric bag filled with 80 grams of Silica gel desiccant was placed in the air circuit, as shown by Figure 1. A 100 g full-scale cantilever strain gauge supported the desiccant bag within the glass sphere. Changes in trapped air moisture content was measured using an air temperature/humidity sensor (Sensirion® SHT75) located within the slave bellows air space.

During the test, air temperature and humidity, as well as silica gel mass data obtained from the cantilever strain gauge was continuously transferred to a PC via a data acquisition board. Additionally, the average values of both air absolute water mass content and water mass absorbed by the silica gel desiccant was recorded every 4 minutes. This time period was sufficient to allow for the slow change in air temperature/humidity and desiccant mass occurring within the test apparatus.

### Post-acquisition data processing

Data was recorded corresponding to the mass of water vapor absorbed from the air by the desiccant along with air water vapor content. Post processing involved the differentiation of this data to determine the rate of change in each parameter with respect to time. Tissue water flux was determined by adding the rate of change in water mass content in the desiccant to that of the air to yield the rate of water efflux from the tissue surface. Maximal water flux values for each test undertaken were then compiled for each pressure setting.

## Results

**Figure 2:**
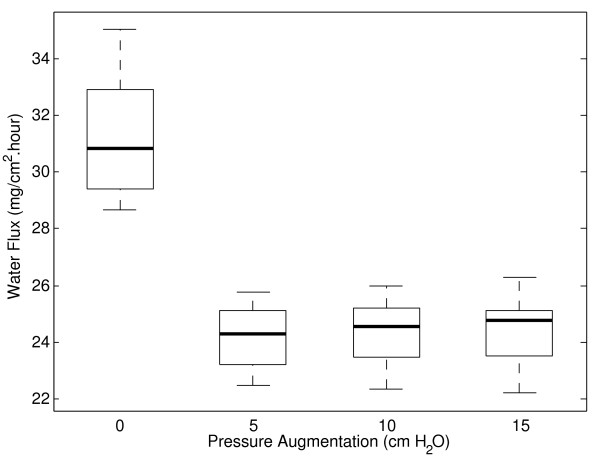
**Trachea maximal water flux results during simulated breathing at ambient and augmented air pressure.** Median values (shown by bold line) demonstrate a 22% reduction in maximal water flux from CPAP pressures of 5 cm H_2_O.

For each tissue test, data corresponding to the amount of water vapor absorbed by the desiccant and air water vapor content along with the sum of the rates of change of both of these variables were measured. The maximal tissue water supply value for all of these experiments over the range of pressures tested is shown by Figure 2. The bold line indicates median value and bottom and top of box are the first and third quartiles respectively. Ends of whiskers indicate minimum and maximum of data (n = 8) at each pressure increment.

Results from this investigation demonstrate a 22% reduction in maximal water flux occurs from 5 cm H_2_O pressure above ambient. To test the hypothesis that pressure augmentation negatively influences epithelial water flux, variation in this parameter was evaluated using a 2-sided linear model (n = 8). This analysis showed a significant difference existed between the ambient and pressurized results (p < 0.0001) and that there was no significant variation between all pressurized results (p = 0.716).

## Discussion

Previous work has shown maximal ASL flux from bovine and porcine tracheal and tracheobronchial tissue under ambient pressure conditions ranges from 10-150 μl/cm^2^.hr [20,23-26]. The results of this study found a maximal water flux of 31.2 μl/cm^2^.hr at ambient pressure which is comparable to that found by the earlier work. This study has also shown a 22% reduction in bovine tracheal mucosa water supply occurs for all pressure augmentations from 5 cm H_2_O. Reduction in ASL supply is possibly due to a decrease in superficial epithelial flux as a result of a constant pressure force/stress adversely influencing the airway cellular purinergic sensory system. While changes in the intraluminal pressure gradient could also influence overall ASL movement, the pressure gradient is unlikely to change between ambient and CPAP breathing. Normally the tidal airflow is regulated by differing airway and pleural pressures. During CPAP therapy there is no significant change in tidal airflow when compared to breathing ambient air so it is unlikely that the intraluminal pressure gradient would change between these two breathing conditions.

The use of desiccant to remove water vapor from the air and create a water demand on the ASL could potentially have influenced the results given its absorption characteristic diminishes with time. To avoid this issue influencing results, care was taken to ensure that each stage of the experiment was undertaken within identical time frames to ensure replication of tissue loading conditions for all tests undertaken. Water flux from the ASL may also have been influenced by changes in water vapor pressure, which is dependent not only upon temperature and pressure, but also changes in chemical concentration. Following Raoult’s Law, an increase in mole fraction of a chemical component within the ASL layer will cause a corresponding reduction in water mole fraction and water vapor pressure, leading to a subsequent drop in water mass flux. Given the chemical composition and concentration is actively regulated by the epithelial cell purinergic system [27], it is unlikely significant variation in water partial pressure would have occurred during the experiment. Changes in air pressure over the range considered by this study are predicted to have caused less than 4% reduction in water vapor concentration to occur. Because of this, neither change in ASL chemical composition or CPAP pressure is likely to have influenced the results of this experiment.

The purpose of this pilot study was to ascertain if ASL supply diminishes during CPAP breathing. During study design we decided that the whole bovine trachea offered a convenient model since it provided a large structural conduit with sufficient surface area for use in the devised test apparatus. Although nasal mucosa is capable of delivering greater water flux when compared to the trachea, both the nose and trachea mucosal tissue are very similar and therefore should exhibit similar pressure elicited ASL water flux behavior. Given the findings of this pilot study, we wish to refine this investigation to more accurately reflect the influence augmented air-pressure has on cultured human nasal epithelial cells during simulated at rest/sleep breathing.

## Conclusions

This pilot study has demonstrated a novel system for exposing airway tissue to tidal conditions and static pressure typically found during ambient and CPAP tidal breathing. It has shown that airway CPAP pressures from 5 cm H_2_O above ambient pressure to 15 cm H_2_O reduced the ASL water supply of bovine tracheal mucosa by 22%. Although the actual mechanism by which this occurs is unclear, these findings provide tracheal mucosa water flux data over the range of augmented pressures normally applied during CPAP therapy. This new finding can be implemented in airway computational models to better predict the occurrence of airway drying. It also justifies the use of supplementary humidification during CPAP treatment. Further work is required looking at the influence pressure has on maximal ASL supply at CPAP pressures less than 5 cm H_2_O.

## Competing interests

None of the authors have a financial relationship with a commercial entity that has an interest in the subject of this manuscript.

## Authors’ contributions

DEW conducted the testing as a doctoral student. RJN and JB mentored DEW. All authors have read and approved this manuscript.

## Authors’ information

DEW is a mechanical engineer who undertook this work as part of a larger program aimed at understanding and modeling nasal drying during breathing under CPAP conditions. RJN is a mechanical engineer specializing in thermodynamics and fluid systems. JB is an Otolaryngologist/Pain Medicine Physician.
